# Is a Perioperative Opioid-Sparing Anesthesia-Analgesia Strategy Feasible in Open Thoracotomies? Findings from a Retrospective Matched Cohort Study

**DOI:** 10.3390/jcm14061820

**Published:** 2025-03-08

**Authors:** Vasileia Nyktari, Georgios Stefanakis, Georgios Papastratigakis, Eleni Diamantaki, Emmanouela Koutoulaki, Periklis Vasilos, Giorgos Giannakakis, Metaxia Bareka, Alexandra Papaioannou

**Affiliations:** 1 School of Medicine, University of Crete, Heraklion, 71003 Crete, Greece; papaioaa@uoc.gr; 2 Department of Anesthesiology, University Hospital of Heraklion, 71110 Crete, Greece; g_stefanakis@yahoo.com (G.S.); papastratigakisg@gmail.com (G.P.); e_diamantaki@yahoo.gr (E.D.); emmanouelakt@yahoo.gr (E.K.); medp2012075@med.uoc.gr (P.V.); 3 Computational Medicine Laboratory (CML), Institute of Computer Science (ICS), Foundation for Research and Technology-Hellas (FORTH), 70013 Crete, Greece; ggian@ics.forth.gr; 4 School of Medicine, University of Thessaly, 41500 Larissa, Greece; barekametaxia@hotmail.com

**Keywords:** thoracotomies, perioperative care, analgesia and anesthesia, nonopioid analgesics, opioid analgesics, postoperative pain, chronic pain

## Abstract

**Background/Objectives**: To assess the feasibility and effectiveness of a perioperative opioid-sparing anesthesia-analgesia (OSA-A) technique without regional nerve blocks compared to standard opioid-based technique (OBA-A) in open thoracotomies. **Methods**: This retrospective, matched cohort study was conducted at a university hospital from September 2019 to February 2021, including adult patients undergoing open thoracotomy for lung or pleura pathology. Sixty patients in the OSA-A group were matched with 40 in the OBA-A group. Outcomes included postoperative pain scores on days 0, 1, and 2; 24-h postoperative morphine consumption; PACU and hospital length of stay; time to bowel movement; and rates of nausea and vomiting. **Results**: Of 125 eligible patients, 100 had complete records (60 OSA-A, 40 OBA-A). Demographics were similar, but ASA status scores were higher in the OBA-A group. The OSA-A group reported significantly lower pain levels at rest, during cough, and on movement on the first two postoperative days, shorter PACU stay, and required fewer opioids. They also had better gastrointestinal motility (*p* < 0.0001) and lower rates of nausea and vomiting on postoperative days 1 and 2. A follow-up study with 68 patients (46 OSA-A, 22 OBA-A) assessing chronic pain prevalence found no significant differences between the groups. **Conclusions**: OSA-A without regional nerve blocks for open thoracotomies is feasible and safe, improving postoperative pain management, reducing opioid consumption, shortening PACU stay, and enhancing early gastrointestinal recovery compared to OBA-A.

## 1. Introduction

Post-thoracotomy pain is severe, multifactorial, and attributed to both nociceptive and neuropathic mechanisms [1,2,3]. The contributing factors include surgical incision, muscular, ligamentous, and neural manipulation, rib retraction or fracture, pleural irritation and thoracostomy tube placement. Failure to control this pain has been linked to increased postoperative pulmonary complications, as it negatively affects deep breathing, coughing, and mobilization [1,2]. Furthermore, thoracic surgical procedures frequently result in persistent pain and increased pain sensitivity, with the reported prevalence of post-thoracotomy pain syndrome (PTPS) ranging from 33% to 91% [1,4,5]. Notably, recent research has identified acute pain following thoracic surgery as a robust predictor for the development of PTPS [6].

Post-thoracotomy pain is commonly addressed by thoracic epidural analgesia, paravertebral blocks, or the administration of opioids intravenously. Thoracic epidural analgesia, the gold standard for post-thoracotomy pain has been associated with significant complications, such as hypotension and epidural hematoma, and with a paradoxical increase in length of stay in hospital [3,7]. Paravertebral blocks are considered equivalent to thoracic epidural analgesia, but they carry the risk of vascular puncture, bleeding, and hematoma formation [8]. Furthermore, while epidural and paravertebral blocks effectively block neurotransmission of intercostal and sympathetic nerves, they do not block the vagus and phrenic nerves [7]. As a result, it is often necessary to add non-steroidal anti-inflammatory drugs or opioids. On the other hand, perioperative use of opioids has been associated with multiple side effects, as well as acute tolerance and hyperalgesia [1,9,10]. At the same time, recent publications have raised concerns regarding the impact of opioids on immunomodulation, immunosuppression, and lung cancer progression [1,11].

Current research indicates a significant gap in conclusive evidence regarding the effects of Opioid Free (OFA-A) and Opioid Sparing (OSA-A) Anesthesia-Analgesia on postoperative outcomes following thoracic surgery. A recent retrospective study on thoracic surgery patients showed a decrease in postoperative pain scores and a concomitant reduction in epidural ropivacaine consumption following the implementation of OFA-A [2].

However, to our knowledge, there is currently no study specifically addressing post-thoracotomy pain management using a perioperative opioid-sparing anesthesia-analgesia strategy (OSA-A) that does not involve a central or peripheral nerve block. The goal of the present study was to examine whether patients receiving perioperative OSA-A or an opioid-based anesthesia-analgesia strategy (OBA-A) for open thoracotomy differed in their postoperative outcomes. Besides pain scores, post-anesthesia unit (PACU) and hospital stay, nausea/vomiting, opioid consumption postoperatively, intestinal mobilization, and persistent postoperative pain were included as study outcomes. Despite conflicting data from the literature, where most evidence suggests that OSA-A/OFA-A protocols do not lower pain scores [10,12,13] our initial hypothesis is that OSA-A group would experience significantly lower pain scores, a shorter duration of stay in the Post-Anaesthesia Care Unit (PACU) and similar morphine equivalent dose (MED) consumption in the immediate postoperative period.

## 2. Materials and Methods

### 2.1. Study Design

This is a retrospective monocentric study conducted at the Anesthesiology Department of the University Hospital of Heraklion, Crete, Greece. The study was conducted in accordance with the Declaration of Helsinki and was approved by the Research Ethics Review Board of the University Hospital of Heraklion (Registration number 14/18-09-2019; Registration date 18 September 2019).

For a long time in our department the standard practice for patients undergoing open thoracotomies had been the combination of general and epidural anesthesia. However, due to an increasing prevalence of anticoagulant use among patients, the option of epidural analgesia had been precluded for several individuals. Consequently, a standard OBA-A technique was performed in these cases, without a regional nerve block. Notably, in February 2016, three consultant anesthesiologists introduced OFA-A/OSA-A for open thoracotomies related to lung or pleural pathology as part of a meticulously audited quality improvement initiative.

This retrospective matched cohort study focused on individuals aged 18 years, American Society of Anesthesiology (ASA) physical status 1–3, who underwent elective open thoracotomy for lung or pleural pathology between February 2015 and February 2021. As the OBA-A technique was used in patients with contraindications to regional nerve blocks (mainly patient refusal or anticoagulation), we decided to implement perioperative OSA-A without the use of any central or regional nerve block; thus, such technique implementation was excluded from the study. Exclusion criteria also included cognitive decline making effective postoperative communication difficult, and incomplete data registration on the patient’s medical record.

Based on previous studies of thoracotomy patients without locoregional techniques [14], we hypothesized that for a reduction of NRS at rest by one point in PACU, we would require 120 patients divided in two groups (60 per group) (a = 0.01, b = 0.10). All patients who underwent thoracic surgery via open thoracotomy from 2015 to 2021 were evaluated for eligibility. Patient matching criteria encompassed type of operation, surgeon, gender and age.

### 2.2. Anesthesia-Analgesia Management

The perioperative management protocols for OBA-A and OSA-A group are summarized in Table 1.

To compare analgesic requirements in the postoperative period, the total amount of morphine (mg) equivalents delivered in the period 0–48 h was recorded. This included PCA morphine (iv), morphine administered in PACU) and any other opioid administered in PACU, on the first (POD1) and second (POD2) postoperative days. All administrated opioids were converted to iv MED according to Table 2 (from iv Morphine equivalents for commonly used drugs—ENCORE study) [15].

### 2.3. Study Outcomes

#### 2.3.1. Pain and Opioid Consumption

Patient pain scores were systematically recorded in a Numerical Rating Scale (NRS) ranging from 0 to 10 in PACU (Postoperative Day 0, designated as POD0), and postoperative days 1 and 2 (POD1, POD2 respectively). Pain assessment in the post-anaesthesia care unit was performed approximately one hour after transfer from the operating theatre, allowing the patient to be fully awake and cooperative.

Cumulative opioid consumption was assessed in terms of intravenous MED over the initial 48 postoperative hours. Opioids were administered under three distinct circumstances: (1) supplemental opioids provided in PACU, (2) patient-controlled analgesia (PCA), and (3) any additional opioids given on ward on POD1 and POD2.

#### 2.3.2. Other Outcomes

In our comparative analysis of patient cohorts, several additional factors were identified as essential: the duration of stay in PACU, the time of bowel function recovery, and the prevalence of nausea/vomiting on POD1 and POD2.

A comprehensive evaluation of persistent pain at the thoracotomy site was conducted during follow-up assessments occurring more than six months postoperatively. To identify the presence of chronic pain, patients were assessed via structured telephone interviews. We ensured that all interviews took place after a minimum of six months following the surgical intervention. All participants provided informed consent for these interviews, which were conducted by a qualified anesthetist. Pain intensity was quantitatively assessed using the Numerical Rate Scale (0–10). Patients were classified as experiencing chronic pain if they reported a score exceeding 0 in response to any of these questions:

1. How would you assess your pain now, at this moment? (0–10)
2. How severe was the worst pain during the past 4 weeks? (0–10)
3. How severe was the pain during the past 4 weeks on average? (0–10)

Chronic pain was classified as moderate, if the patients reported NRS of 4–6 in any of the above questions and as severe if they reported a score ≥ 7 to any of the above questions. Neuropathic pain characteristics were detected by using the PainDETECT questionnaire [16].

### 2.4. Statistical Analysis

Categorical data were analyzed using two-sided Fisher’s exact test, to check if there are significant associations between categorical parameters. Q-Q plots and Kolmogorov-Smirnov normality tests were used to determine numerical data normality. Statistical differences between groups (OBA-A, OSA-A) were evaluated using independent samples *t*-test for normally distributed data and Mann-Whitney U test otherwise. To assess potential differences of factors (such as morphine consumption and pain data) that were measured at multiple time points, since these data were found to be non-normally distributed, multiple Mann-Whitney U tests were used and adjustments for multiple comparisons were made using the False Discovery Rate (FDR) method (Q = 1%). Numerical data were reported as $\bar{x}$ ± SD, and the reported *p*-values were corrected using the FDR method as mentioned. Data were analyzed using GraphPad Prism 10 (edition 10.3.1 (509)) (GraphPad Software, Boston, MA, USA).

## 3. Results

### 3.1. Patients’ Characteristics

**Figure 1:**
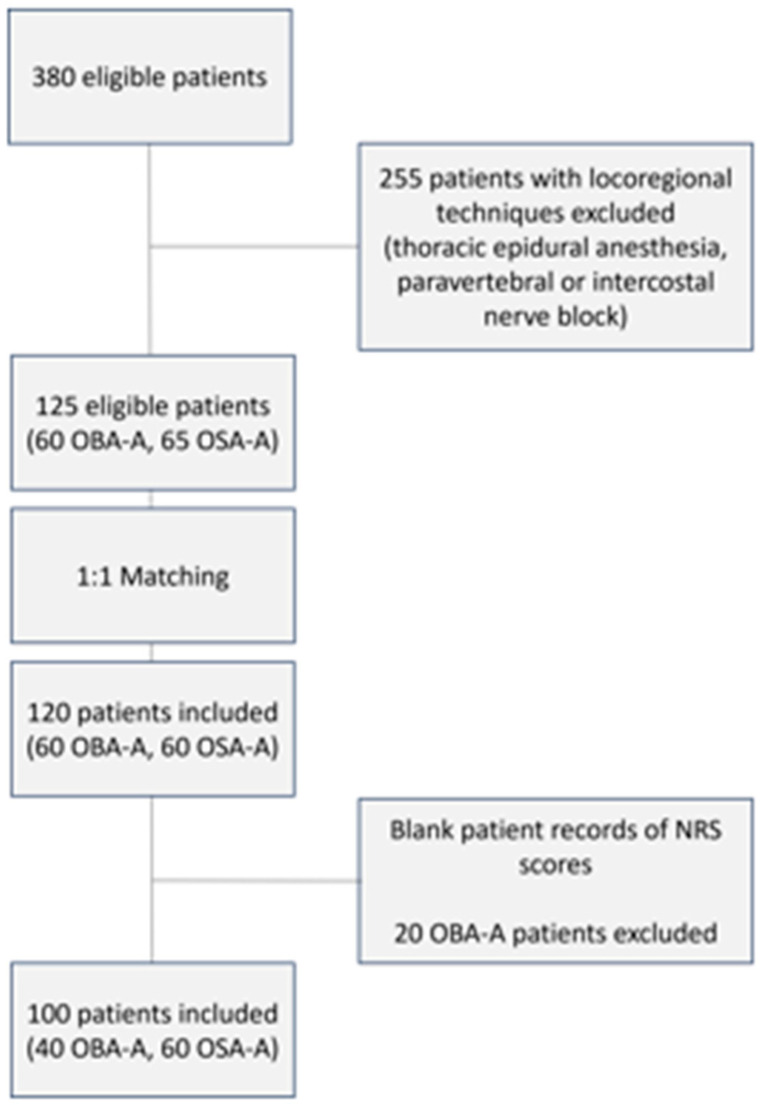
Flow chart of patients’ recruitment.

We have conducted a retrospective analysis of the acute postoperative pain service records in our hospital from 1 February 2015 to 1 February 2021. Among 380 thoracic surgeries for lung and pleura pathology, we identified 125 eligible patients for the study where locoregional techniques had not been performed, 60 in the OBA-A group and 65 in the OSA-A group (Figure 1). After matching the patients, each group included 60 patients. However, due to incomplete records, only 100 of these 120 patients had comprehensive data, including demographic information, intraoperative pharmacological agents’ administration, PACU data, and most importantly details regarding the first 2 postoperative days (60 patients in the OSA-A group and 40 patients in the OBA-A group). Our attempts to recruit additional patients for the OBA-A group by expanding the study’s period backwards, were unsuccessful because no patient postoperative pain records were found. Having undergone a thoracotomy protocol shift in our department, since 2016 as mentioned, we had no OBA-A patients moving forward from that period.

As indicated in Table 3, patients did not differ in basic demographic characteristics such as age, sex, weight, height and BMI. The only identified difference pertained to the fact that patients in the OBA-A group had higher ASA status scores.

### 3.2. Primary Outcome

#### 3.2.1. Pain Assessment

The patients’ pain levels were assessed on NRS at three different time points: POD0 (during stay in PACU), POD1 and POD2. Data from both groups showed a non-normal distribution and thus two-tailed multiple Mann-Whitney U tests were used.

#### 3.2.2. Pain Score at Rest

**Figure 2:**
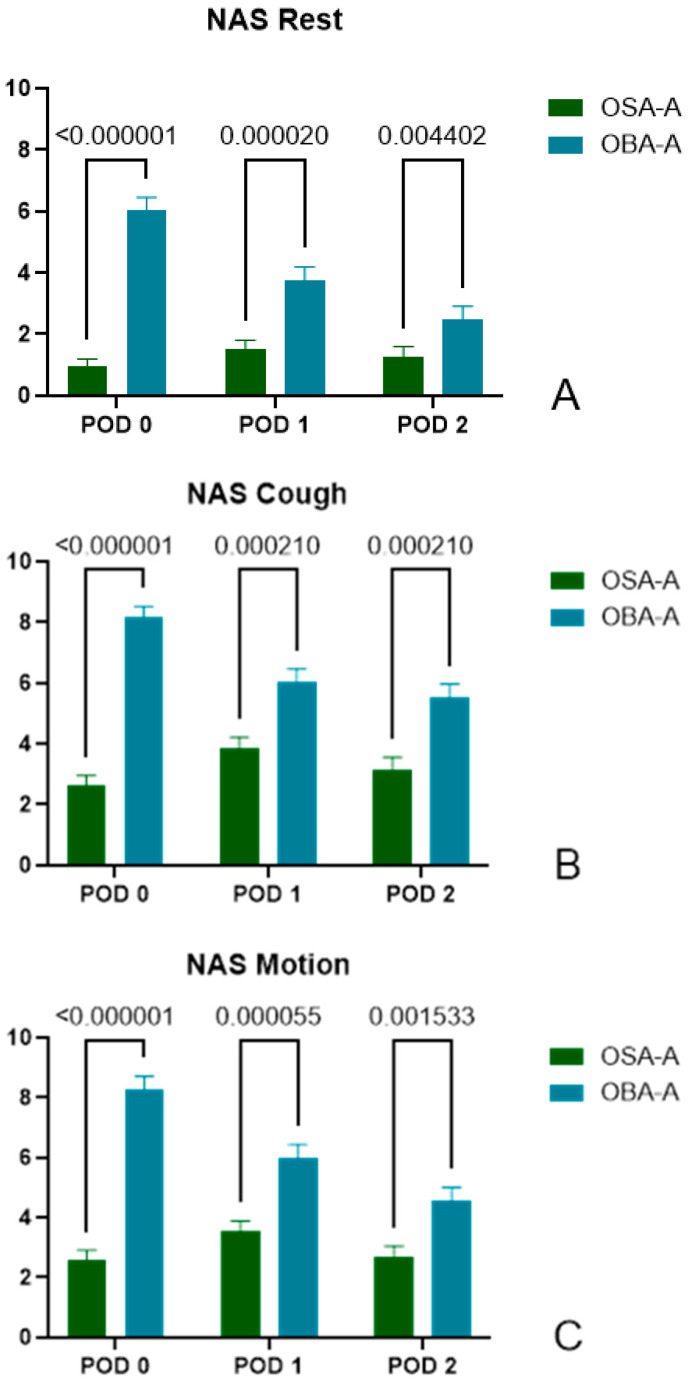
Pain scores during rest (**A**) NRS rest, cough (**B**) (NRS cough) and motion (**C**) (NRS motion) in PACU (POD0), POD1 and POD2. Note that the OSA-A group reported significantly lower levels of pain at all-time points.

As shown in Figure 2A, patients in the OSA-A group reported significantly lower pain levels during rest at all time points, POD0, POD1, and POD2.

- POD0: (OSA-A) 0.95 ± 1.92, *n* = 60 vs. (OBA-A) 6.05 ± 2.56, *n* = 40, *p* < 0.000001.
- POD1: (OSA-A) 1.53 ± 2.12, *n* = 57 vs. (OBA-A) 3.78 ± 2.66, *n* = 40, *p* = 0.000020.
- POD2: (OSA-A) 1.28 ± 2.34, *n* = 54 vs. (OBA-A) 2.47 ± 2.57, *n* = 34, *p* = 0.004402.

#### 3.2.3. Pain Score During Cough

Notably, patients in the OSA-A group reported significantly lower levels of pain during cough on POD0, POD1 and POD2.

- POD0: (OSA-A) 2.62 ± 2.67, *n* = 60 vs. (OBA-A) 8.18 ± 2.14, *n* = 39, *p* < 0.000001.
- POD1: (OSA-A) 3.84 ± 2.83, *n* = 57 vs. (OBA-A) 6.03 ± 2.78, *n* = 40, *p* = 0.000210.
- POD2: (OSA-A) 3.15 ± 2.99, *n* = 54 vs. (OBA-A) 5.51 ± 2.65, *n* = 35, *p* = 0.000210.

#### 3.2.4. Pain Score During Motion

Patients in the OSA-A group reported significantly lower levels of pain during motion, on POD0, POD1 and POD2.

- POD0: (OSA-A) 2.58 ± 2.49, *n* = 60 vs. (OBA-A) 8.29 ± 2.65, *n* = 38, *p* < 0.000001.
- POD1: (OSA-A) 3.54 ± 2.61, *n* = 57 vs. (OBA-A) 5.98 ± 2.92, *n* = 40, *p* = 0.000055.
- POD2: (OSA-A) 2.69 ± 2.70, *n* = 54 vs. (OBA-A) 4.54 ± 2.76, *n* = 35, *p* = 0.001533.

### 3.3. Secondary Outcomes

#### 3.3.1. PACU and Hospital Length of Stay

Patients in the OSA-A group had a significantly shorter PACU stay, measured in hours (OSA-A group 2.030 ± 1.022 vs. OBA-A group 3.125 ± 1.036 (*p* < 0.0001) (see Table 4). No difference in hospital length of stay, measured in days, was observed between the two groups (OSA-A group 5.77 ± 3.45 vs. OBA-A group 5.73 ± 2.05, *p*: 0.2669).

#### 3.3.2. Rescue Analgesia and Morphine Consumption in PACU and on POD1 and POD2

Patients in the OBA-A group requested analgesic interventions in PACU frequently, including both opioids and non-opioids. Specifically, a total of 27 patients in the OBA-A group required analgesic administration, whereas 21 patients in the OSA-A group required such intervention, resulting in a statistically significant difference (*p* = 0.0021).

**Figure 3:**
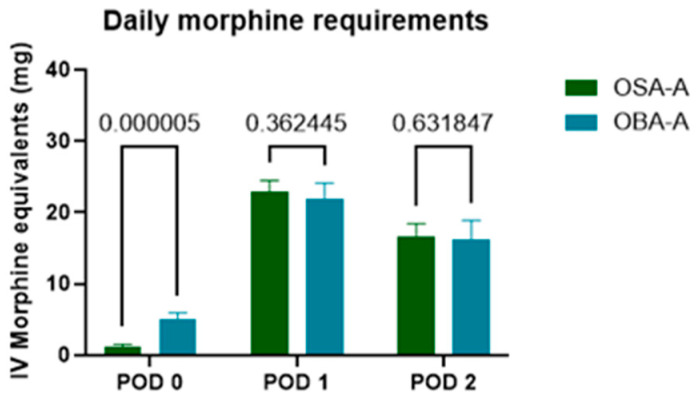
Comparison of analgesic requirements between the OSA-A and OBA-A groups, measured in MED, in PACU (POD 0) and on POD 1 and POD 2. Notably, a significant reduction in analgesic requirement was observed only during the PACU stay.

During their stay in PACU (POD 0), OSA-A patients were administered tramadol for rescue analgesia, in an average dosage of 1.177 ± 2.929 mg of MED. In comparison, the OBA-A group received an average of 5.031 ± 5.705 mg of MED (*p* = 0.000005). This significant reduction in opioid consumption was not sustained in the subsequent postoperative days. On POD1 the OSA-A group demonstrated an average consumption of 22.86 ± 12.76 mg of MED while the OBA-A group reported an average of 21.88 ± 14.29 mg (*p* = 0.362445 two-tailed). On POD 2, the OSA-A group required an average of 16.54 ± 14.72 mg of MED, in contrast to the OBA-A group’s average of 16.23 ± 16.66 mg (*p* = 0.631847) (see Figure 3, Table 4).

#### 3.3.3. Gastrointestinal Motility

Our findings indicate that patients within the OSA-A group exhibited a substantially higher incidence of intestinal motility on POD1 and POD2. Intestinal mobility (defined as occurrence of flatus, successful bowel movements or presence of bowel sounds) returned in more patients in OSA-A group in POD1 (51 vs. 8, *p* < 0.0001) and POD2 (58 vs. 22, *p* < 0.0001) in comparison to OBA-A patients (see Table 4).

#### 3.3.4. Nausea-Vomiting

Patients in the OSA-A group reported significantly lower rates of postoperative nausea and vomiting in the first two postoperative days (see Table 4).

#### 3.3.5. Chronic Pain

We successfully communicated with 68 patients to assess chronic pain following thoracotomy. Among them, 46 participants were in the OSA-A group, while 22 were in the OBA-A group. Participants in the OSA-A group were evaluated significantly earlier than those in the OBA-A group, with a mean assessment timespan of 2.52 ± 0.69 years for the OSA-A group compared to 4.41 ± 0.85 years for the OBA-A group (*p* < 0.0001).

27 patients reported chronic pain in varying intensity, 18 in the OSA-A group and 9 in OBA-A group. No statistically significant differences were observed between the two groups regarding prevalence or severity of chronic pain. Neuropathic pain was not confirmed in any patient. Only 5 were categorized as “uncertain” based on the pain detection questionnaire: 3 in the OSA-A group and 2 in the OBA-A group. Again, no significant differences were observed between the 2 groups.

## 4. Discussion

This retrospective study aimed to evaluate the effectiveness of an opioid-sparing analgesic strategy in comparison to a standardized opioid-based approach for patients undergoing conventional open thoracotomy due to lung or pleural pathology, when regional blocks were not performed. Our findings revealed that patients in the OSA-A group reported significantly lower pain levels during rest, coughing, and movement on the day following surgery and throughout the first two postoperative days, shorter PACU stay, less nausea and vomiting and earlier recovery of gastrointestinal motility.

Acute postoperative pain presents a complex and multifaceted challenge, characterized by significant variability among patients [17]. The perception of pain arises from the interplay of various factors, including noxious stimuli, peripheral nerve transmission, and central processing of pain signals. Furthermore, the effectiveness of both pharmacological and non-pharmacological interventions is significantly influenced by individuals’ cognitive interpretations of their pain experiences.

In this context, the optimal pain management protocol following thoracic surgery has yet to be established. It is critical to consider both the specific type of surgery performed—such as Video-Assisted Thoracoscopic Surgery (VATS) or open thoracotomy—and the individual characteristics of each patient, including pulmonary function, chronic pain history, and any additional comorbidities. Thoracic surgery can impair pulmonary function, and this impairment may be exacerbated by pain [1,2,18]. For these reasons, it is imperative to implement an analgesic protocol that incorporates multimodal and regional analgesia, tailored to meet each patient’s unique needs and preferences.

Multimodal analgesia is recognized as the standard of care for pain management in thoracic surgery and integrates regional anesthetic techniques, including epidural anesthesia and fascial plane blocks, with oral regimens that either utilize opioids or prioritize opioid-sparing options [1,19]. The PROSPECT thoracotomy subgroup advocates for the perioperative application of regional analgesia, specifically paravertebral block or thoracic epidural, based on robust evidence derived from randomized controlled trials (presented in the Procedure Specific Postoperative Pain Management, www.postoppain.org (accessed on 20 December 2024)). Additionally, the PROSPECT recommendations do not support the perioperative use of gabapentinoids, ketamine, or dexmedetomidine in open thoracotomies, due to inconsistent evidence. If a paravertebral block or thoracic epidural is not feasible due to anticoagulation or patient preference, the use of strong systemic opioids alongside non-opioid analgesia is recommended as a rescue measure.

However, this raises the question: does the inability to use regional techniques mean that opioid-free (OFA) or opioid-sparing (OSA) anesthesia is not feasible in open thoracotomies? Thoracic surgery is a significantly challenging field associated with a higher rate of pain and pulmonary complications compared to other types of surgery. Experience in OFA/OSA techniques gained in other surgical fields probably cannot be directly applied to thoracic surgery. In the first systematic review and meta-analysis of opioid-free anesthesia in thoracic surgery by D’Amico et al., where the primary outcome was the occurrence of any postoperative complication, six studies were included as relevant [12]. In reviewing the OFA/OSA protocols all studies incorporated either paravertebral block (PVB), serratus anterior plane block (SAPB) or erector spinae plane block (ESPB). Patients in the OFA group exhibited a significant reduction in the postoperative complication rate compared to OBA. PACU length of stay was similar between the two groups and better analgesia with less opioid consumption at 48 h after surgery was observed in the OFA group.

The significance of a perioperative strategy that minimizes or eliminates opioid use in thoracic surgery on patient outcomes is emphasized in a recent retrospective study by J. Xiao et al. In their study, the authors conclude that the amount of opioid medication received during hospitalization was an independent risk factor for postoperative complications [10]. Additionally, several studies have demonstrated a correlation between intraoperative and postoperative opioid administration, the development of chronic pain, opioid addiction, and patient survival [11,20,21,22,23].

In our study, the lower levels of reported pain within the OSA-A group may be attributed to the perioperative use of agents affecting both nociceptive and neuropathic elements of pain, such as pregabalin, dexmedetomidine, ketamine, lidocaine, tramadol and magnesium sulphate [24,25,26,27]. It is essential to recognize that pain after thoracotomy may arise from both nociceptive and neuropathic mechanisms, with opioids frequently demonstrating limited efficacy in the alleviation of neuropathic pain [24,25]. In the study by Homma et al., the preoperative use of hypnotics, thoracotomy, and a duration of surgery ≥ 2.5 h were associated with an increased risk of neuropathic pain post-thoracic surgery [27]. If neuropathic pain is left untreated, it interferes with sleep and is associated with depression and anxiety that will negatively influence the responses to analgesic drugs [24].

First-line drugs for neuropathic pain include antidepressants (tricyclic antidepressants and serotonin–noradrenaline reuptake inhibitors) and anticonvulsants acting at calcium channels (pregabalin and gabapentin). Second- and third-line drugs for neuropathic pain include topical lidocaine and opioids [24]. Opioids are not considered as first choice agents, because of adverse drug reactions and, more recently, because of concerns about abuse, diversion, and addiction [25,28]. Furthermore, the use of intraoperative opioids may be linked to postoperative hyperalgesia and tolerance, resulting in elevated pain scores and increased opioid consumption [10,29,30].

Our results do not agree with those of a recent retrospective study by Devine et al., where no significant differences in referred pain scores were found within the first 24 h after surgery, when comparing an OFA to a standard opioid-based (OBA) technique [30]. It is important to note that their protocols differed from ours, as regional blocks were utilized in both groups. Their OFA technique included intravenous lidocaine (0.75–1 mg/kg) and magnesium sulfate (0.05–0.1 mmol/kg) with propofol and a paravertebral block. Their OBA technique involved intraoperative fentanyl, remifentanil, and/or morphine for analgesia, plus paracetamol and parecoxib when appropriate. All patients received morphine patient-controlled analgesia (PCA) for the first 24 h.

Our protocol incorporates the perioperative use of pregabalin in a dose tailored to patient needs and assessment. Pregabalin is an analogue of γ-aminobutyric acid that binds to the α2-δ subunits of voltage-gated calcium channels in the central nervous system. When used in a perioperative setting, pregabalin can help prevent the development of pain by inhibiting presynaptic voltage-gated calcium channels, which are involved in central sensitization [31]. Pregabalin was preferred to gabapentin due to its more established anxiolytic and analgesic effects and safer profile [32]. Pregabalin has linear pharmacokinetics, dose adjustment is easier, analgesic effects occur faster than gabapentin and exhibits positive effects on anxiety and sleep [33].

Pregabalin was administered preoperatively, on the evening prior to surgery and again on the morning of the procedure, in a dosage ranging from 25 mg to 150 mg. Postoperatively, the administration of pregabalin continued throughout the duration of hospitalization and was extended for the first month after surgery. A study conducted by Homma et al. reported a significant preventive effect on postoperative neuropathic pain in patients who received 25 mg of oral pregabalin twice daily from postoperative day 2 (POD 2) through three months following lung resection [34]. Additional studies have demonstrated the efficacy of perioperative administration of pregabalin for pain management after thoracic surgery [35,36,37]; however, the results have yet to achieve a consistent level of acceptance across the literature. In a very recent meta-analysis by Zhang and Zhang, data from five RCTs and 329 patients undergoing thoracic surgery confirmed that pregabalin was effective to significantly reduce pain at 24 h and the incidence of persistent neuropathic pain [38]. The authors comment that the doses and use of pregabalin in the various studies were not completely the same, which may have caused some heterogeneity. In this context, the decisions regarding when to start treatment, the initial dosage, titration protocols, and the suggested duration of administration are still unclear. Since higher dosages, particularly those exceeding 150 mg, are linked to increased sedation levels, we have chosen to begin treatment with an initial dosing titration range of 25 mg to 150 mg. In selecting the appropriate dosage, we carefully considered various factors, including the patient’s age, weight, and overall health assessment. Additionally, we made necessary dose adjustments for patients classified as ASA-3 and for individuals identified as underweight, specifically those with a Body Mass Index (BMI) of less than 20. The analgesic efficacy of ketamine has been well established in various surgical contexts [39,40,41]. In addition to its well-known analgesic effects, primarily mediated by NMDA receptor antagonism, ketamine exhibits several other beneficial effects, including anti-inflammatory and anti-hyperalgesic properties [40]. Administering an initial bolus of ketamine at the onset of anesthesia, followed by an intraoperative infusion or additional boluses, has been shown to significantly improve postoperative pain control, while it also reduced the need for opioids by 30–40% [39]. It can be administered through several routes, including oral, intravenous, epidural, and wound infiltration. The dosing of ketamine also varies, ranging from single-dose boluses of up to 1 mg/kg to continuous iv infusions of up to 0.18 mg/kg/h for 48 h postoperatively [39]. In our protocol, we routinely administered ketamine in two boluses: the first during anesthesia induction at a dose of 0.5–1 mg/kg, and the second 20 min before surgical closure at a dose of 20–30 mg. These doses are considered low for postoperative pain management (defined as no more than 1.2 mg/kg/h when used as a continuous infusion and no more than 1 mg/kg when given as a bolus) [42].

Magnesium sulphate (MgSO_4_) has been shown to produce an antinociceptive effect on animal models of neuropathic and inflammatory pain [43]. It possesses opioid-sparing effects by blocking the NMDA receptor and central sensitization, similarly to ketamine [43,44]. A recent metanalysis of randomized trials supported the perioperative use of magnesium as an analgesic adjuvant at 24 h following thoracic surgery, but no opioid-sparing effect at 48 h post-surgery was exhibited [45]. In the study of Abdelgalil et al. the combined preoperative single dose of pregabalin and magnesium sulfate was associated with reduced postoperative pain and total morphine consumption in patients undergoing thoracotomy [46].

Dexmedetomidine is a highly selective α2-adrenergic agonist known for its ability to inhibit norepinephrine release, thereby suppressing sympathetic activity within the central nervous system [47,48]. It exhibits analgesic and sedation properties that have increasingly been used to reduce postoperative opioid consumption [49,50,51]. In the context of thoracic surgery, propensity-matched studies examining intraoperative dexmedetomidine infusions have demonstrated no significant differences in pain scores or opioid consumption throughout the hospital stay [52,53]. These varying outcomes indicate that discrepancies in the effectiveness of dexmedetomidine-based opioid-sparing analgesia may arise from differences in administration protocols, including the use of adjunct medications, dosing strategies, timing of infusions, and the characteristics of patient populations.

Post thoracotomy pain syndrome (PTPS) is characterized by an aching sensation in the distribution of the incision, and it usually resolves within 2 months postoperatively [54]. Pain that persists or recurs for longer than 3 months after thoracic surgery is defined as chronic post-thoracotomy pain (CPTP). Its reported prevalence rates range from 27% to 47% and many survivors of lung cancer experience a substantial decline in their functional capacity and overall quality of life because of CPTP [5,54,55,56,57,58,59,60,61,62]. The World Health Organization included detailed diagnostic criteria in the 11th revision of the International Classification of Disease (ICD-11), where CPTP was defined as any unpleasant or painful sensation persisting or recurring more than three months after surgery in relation to the operation area [63].

Open thoracotomy is associated with higher risk of CPTP [64]. Compared with nociceptive pain, neuropathic pain is claimed to be the major cause of PPSP after breast and thoracic surgeries [64,65]. In our study 68 patients (46 belonging to the OSA-A group and 22 to the OBA-A group) were assessed for CPTP. Ιn accordance with the literature, 27 patients reported chronic pain (39.7%). Noteworthy, no patient in both cohorts was diagnosed with neuropathic pain, using the PainDETECT questionnaire.

## 5. Limitations

This study has several potential limitations, primarily stemming from its retrospective nature and the characteristics of the data sources used. As a retrospective study, it was influenced by the lack of blinding for the anesthesiologists in both groups, which introduces the possibility of bias. Another significant drawback of this study design was the limited control over the sampling of the population. A major issue we encountered was missing data, which posed substantial challenges in our analysis. The initial one-to-one matching was not feasible due to incomplete medical records. Furthermore, our attempts to recruit additional patients for the OBA-A group in recent years have uncovered problems within the hospital’s registry. Parameters that were important or relevant were not recorded in the health record. Confounding variables and causality are difficult to prove, as in all observational studies.

Despite these limitations, the retrospective design reflects real-life clinical practice, which contrasts with randomized controlled trials (RCTs) that typically involve highly selected patient populations enrolled under strict eligibility criteria.

## 6. Conclusions

Our findings support the use of a multimodal, opioid-sparing perioperative anesthesia and analgesia strategy for patients undergoing open thoracotomies, which could be considered in situations where a regional nerve block or thoracic epidural are not feasible due to anticoagulation or patient refusal. Implementing this protocol in our patients resulted not only in improved pain control but also in a better quality of recovery. Further well-designed prospective randomized studies are necessary to clarify specific opioid-free or opioid-sparing perioperative protocols that will enhance pain management and improve patient outcomes.

## Figures and Tables

**Table 1:** Perioperative protocols in OBA-A and OSA-A groups.

	OBA-A Group	OSA-A Group	Comments
Premedication Night before surgery	Anxiolysis Bromazepam 1.5 mg orally (po)	Pregabalin 25–150 mg po Amitriptyline 10 mg po	Pregabalin tailored to patients’ needs/status Low-dose Amitriptyline for its antinociceptive and anti-salivary actions, if not contraindicated due to patients’ comorbidities
Premedication Day of surgery	Midazolam 0.05 mg/kg intramuscularly (im)	Pregabalin 25–150 mg po Amitriptyline 10 mg po	
Anesthesia Induction	intravenously (iv) iv Fentanyl 150–200 mcg	iv Dexmedetomidine loading dose 1 mcg/kg administrated over 15 min	
	iv Propofol 2–2.5 mg/kg	iv Midazolam 2–5 mg	
	iv Rocuronium 0.6 mg/kg (1.2 mg/kg if RSI)	iv Ketamine 0.5–1.0 mg/kg	
	Or	iv Propofol 1–2 mg/kg	
	iv Cis-Atracurium 0.2 mg/kg	iv Lidocaine 1 mg/kg	
	* Surgical site infiltration prior to incision with Ropivacaine (10–15 mL Ropivacaine 0.75%)	iv Rocuronium 0.6 mg/kg (1.2 mg/kg if RSI)	
		iv Magnesium Sulphate 30–40 mg/kg as bolus over 15 min	
		iv Dexamethasone 8–16 mg	
		* Surgical site infiltration prior to incision with Ropivacaine (10–15 mL Ropivacaine 0.75%)	
Prior to incision	iv Fentanyl 50–100 mcg	iv Dexmedetomidine infusion 0.6–1.2 mcg/kg/h	
	Or	iv Lidocaine infusion 1 mg/kg/h	
	iv Remifentanil infusion 0.05–0.25 mcg/kg/h	iv Paracetamol 1 gr	
		iv NSAID (Parecoxib 40 mg or Dexketoprofen 50 mg)	
Anesthesia maintenance	Volatile anesthesia	Volatile anesthesia	
Intraoperative antinociception	iv Remifentanil infusion 0.05–0.25 mcg/kg/h	iv Lidocaine infusion 0.5–1 mg/kg/h	
	Or	iv Dexmedetomidine 0.4–1 mcg/kg/h	
	iv boluses of Fentanyl 50–100 mcg as required	iv boluses of 20–30 mg of Ketamine as required	
		iv bolus of 2.5 g Magnesium sulphate	
20 min prior to surgical closure	iv NSAID if no contraindication (Parecoxib 40 mg or Dexketoprofen 50 mg)	iv bolus of 20–30 mg of Ketamine	
	iv Paracetamol 1 g	iv Tramadol 100 mg	
	iv Morphine 0.05–0.15 mg/kg		
PACU	iv bolus of Morphine 2 mg	iv Ketamine 30–50 mg	If patient’s pain score at rest ≥6 in the numerical rating scale score 0–10
		± iv Magnesium sulphate 2.5 g	
		± iv Midazolam 1–2 mg	
	iv Pethidine 20–30 mg	iv Pethidine 20–30 mg	If shivering
		iv Tramadol 100 mg	Rescue therapy
Surgical Ward 48 h after surgery	iv Paracetamol 1 gr every 8 h	iv Paracetamol 1 gr every 8 h	
	iv PCA Morphine (Morphine solution 0.5 mg/mL) with an infusion rate 0.5–1 mg/h and possibility of bolus 1 mg every 10 min, if no contraindication, under continuous monitoring with pulse oximetry	iv Tramadol (max daily dose 300 mg)	
		po Pregabalin 25–150 mg daily dose, given in titrated doses	
		Rescue therapy: im Pethidine 50–75 mg	

* In the OBA-A group, the option of wound infiltration with ropivacaine was available prior to incision, according to surgeon preference, as per our department’s protocol. Thus, the same option was also available for subsequent patients in the OSA-A group.

**Table 2:** Opioid conversion table used to equate equivalent iv morphine values [15].

Total Daily Dose (IV)	Morphine Equivalent Dose (mg)
1 mcg fentanyl iv	0.066 mg morphine iv
1 mg oxycodone iv	1.5 mg morphine iv
1 mg tramadol	0.1 mg morphine iv
1 mg pethidine iv	0.13 mg morphine iv

**Table 3:** Patients’ characteristics.

Characteristic		OSA-A ($\bar{x}$ ± SD)	OBA-A ($\bar{x}$ ± SD)	*p*-Value	Statistical Test
Age (y)		62.55 ± 11.29	63.73 ± 12.55	0.6270	Unpaired two-tailed *t* test
Weight (kg)		79.50 ± 15.33	77.43 ± 13.28	0.4864	Unpaired two-tailed *t* test
Height (m)		1.698 ± 0.07095	1.678 ± 0.08057	0.1996	Unpaired two-tailed *t* test
BMI (kg ∗ m^−2^)		27.56 ± 5.050	27.40 ± 3.751	0.8658	Unpaired two-tailed *t* test
Sex	Male	48	28	0.3394	Fisher’s exact test two-sided
	Female	12	12		
ASA physical status	1	0	1	0.0208	Fisher’s exact test two-sided
	2	39	16		
	3	21	22		
	4	0	1		
Type of surgery	Lobectomy	39	23	0.3728	Fisher’s exact test two-sided
	Segmentectomy	14	12		
	Pneumonectomy	3	0		
	Other (biopsy, talc pleurodesis etc.)	4	5		
Depression	Yes	7	7	0.5576	Fisher’s exact test two-sided
	No	53	33		
Anxiety	Yes	20	11	0.6599	Fisher’s exact test two-sided
	No	40	29		
Alcohol Use Disorder	Yes	6	1	0.2375	Fisher’s exact test two-sided
	No	54	39		
Preoperative chronic pain medication use	Yes	13	3	0.0930	Fisher’s exact test two-sided
	No	47	37		
Preoperative chronic opioid use	Yes	3	1	0.6479	Fisher’s exact test two-sided
	No	57	39		
Preoperative Steroid use (6 months)	Yes	5	0	0.0813	Fisher’s exact test two-sided
	No	55	40		

BMI: Body Mass Index; ASA physical status: American Society of Anesthesiologists Physical Status.

**Table 4:** Secondary outcomes in OSA-A and OBA-A groups.

Secondary Outcome		OSA-A ($\bar{x}$ ± SD)	OBA-A ($\bar{x}$ ± SD)	*p*-Value	Statistical Test
Length of stay PACU (h)		2.030 ± 1.022	3.125 ± 1.036	<0.0001	Mann-Whitney U test
Length of stay Hospital (d)		5.767 ± 3.451	5.725 ± 2.050	0.2669	Mann-Whitney U test
Analgesics Requested PACU (POD 0)	Yes	21	27	0.0021	Fisher’s exact test
	No	39	13		
Analgesics Requested POD 1	Yes	3	2	>0.9999	Fisher’s exact test
	No	57	38		
Analgesics Requested POD 2	Yes	1	0	>0.9999	Fisher’s exact test
	No	59	40		
Morphine (mg) Equivalents Delivered	PACU	1.177 ± 2.929	5.031 ± 5.705	0.000005	Multiple Mann-Whitney U tests correcting for multiple comparisons by using the FDR method
	POD1	22.863 ± 12.760	21.878 ± 14.288	0.362445	
	POD2	16.542 ± 14.723	16.234 ± 16.661	0.631847	
Intestinal Mobilization POD 1	Yes	51	8	<0.0001	Fisher’s exact test
	No	9	32		
Intestinal Mobilization POD 2	Yes	58	22	<0.0001	Fisher’s exact test
	No	2	18		
Nausea & Vomiting POD 1	Yes	1	5	0.0363	Fisher’s exact test
	No	59	35		
Nausea & Vomiting POD 2	Yes	0	4	0.0204	Fisher’s exact test
	No	60	34		

## Data Availability

The data on which this retrospective study is based can be obtained from the corresponding author upon reasonable request, provided that the copyrights of the respective authors are respected.

## Abbreviations

The following abbreviations are used in this manuscript:

OSA-A	Opioid-Sparing Anesthesia-Analgesia
OBA-A	Opioid-Based Anesthesia-Analgesia
PACU	Post Anesthesia Care Unit
PTPS	Post-Thoracotomy Pain Syndrome
MED	Morphine Equivalent Dose
ASA	American Society of Anesthesiology
RSI	Rapid Sequence Induction
NSAID	Non-Steroidal Anti-Inflammatory Drug
PCA	Patient Controlled Analgesia
POD	Post-Operative Day
NRS	Numerical Rate Scale
ANOVA	ANalysis Of Variance
FDR	False Discovery Rate
BMI	Body Mass Index
VATS	Video-Assisted Thoracoscopic Surgery
OFA	Opioid-Free Anesthesia
OSA	Opioid-Sparing Anesthesia
OBA	Opioid-Based Anesthesia
PVB	ParaVertebral Block
SAPB	Serratus Anterior Plane Block
ESPB	Erector Spinae Plane Block
RCT	Randomized Controlled Trial
NMDA	N-Methyl-D-aspartic Acid
CPTP	chronic post-thoracotomy pain
ICD	International Classification of Disease
